# Personality traits and investor profile analysis: A behavioral finance study

**DOI:** 10.1371/journal.pone.0214062

**Published:** 2019-03-27

**Authors:** Daiane De Bortoli, Newton da Costa, Marco Goulart, Jéssica Campara

**Affiliations:** 1 Department of Economics, Federal University of Santa Catarina, Florianopolis, SC, Brazil; 2 Business School, Pontifical Catholic University of Parana, Curitiba, PR, Brazil; 3 Department of Business Administration, Federal University of Santa Catarina, Florianopolis, SC, Brazil; 4 Department of Business Administration, Santa Catarina State University, Florianopolis, SC, Brazil; The Bucharest University of Economic Studies, ROMANIA

## Abstract

This study investigates which of four paradigms best portrays the risk profile manifest by investors in their financial asset investment decisions. The paradigms used to explain this profile were: prospect theory, investor profile analysis (IPA), the Big Five Personality Test, and the Cognitive Reflection Test (CRT). The choice of proxy for the risk preferences (profile) of a typical investor was defined by simulating investments in a laboratory setting. The results are analyzed using ordered logistic regression and show that people who have greater risk tolerance according to IPA, who violate prospect theory, and who have a high degree of openness to experience have the greatest probability of taking higher levels of risk in their investment decisions. With regard to the CRT, higher numbers of correct responses in this test has an inverse relationship with risk taking.

## Introduction

Modern financial theory is based on the concept of *homo economicus*, adopted from neoclassical economics. This ideal, self-interested, and perfectly rational agent maximizes his utility by choosing at each point in time the best options available. This perfect rationality, combined with the efficient markets hypothesis, was assumed by [1] when he developed his portfolio selection theory, which is considered the starting point of modern finance theories. The market efficiency concept was formally set out by [2] and modern financial theories are founded on the assumptions of rational investors and efficient markets.

In contrast, the *agent* of behavioral finance is not perfectly rational, but a normal human who acts and takes decisions under the influence of emotions and cognitive errors [3]. The confirmation of such evidence emerged from [4], from which interdisciplinary elements (in particular from psychology) began to be incorporated into behavioral theories of finance, in attempts to understand the process of decision-making under risk.

Subsequently, several efforts were made to understand which factors could influence the behavior of economic agents. Personality traits, according to [5], are an aspect of this research, since they can determine how each individual processes the information in the market and thereby change their relationship to risk. Realizing the relationship between personality and financial decisions, [6] incorporated into their study personality traits. The authors revealed that extroverted and/or empathic individuals have a greater tendency to follow the behavior of the others in investment decisions, that is, to present herd behavior. Similarly, [7] reported that extroverted, adventurous, and open to new experiences individuals tend to make more rational financial decisions in the face of biases like the certainty effect, the sunk cost fallacy, and mental accounting. Contrary results also indicate that personality may not be significant for financial decisions [5].

These studies show the importance of evaluating the influence of personality traits on investment decisions. However, they assess the behavior of economic agents in a general risk environment, and it is important to evaluate whether such factors could change the risk profile. In addition, factors other than personality traits may also be relevant to explain the risk profile of investors, such as behavioral biases, risk profile questionnaires and cognitive ability [8, 9, 10].

For example, [8] evaluated personality traits and found that individuals who were more open to new experiences and extroverts tended to be more risk-prone. Regarding behavioral biases, [9] shows that behavioral biases such as overconfidence and myopia (often checking their financial performance) are good indicators for risk profile. However, he has shown that questionnaires adopted by banks to determine the risk profile of investors are not good predictors of the real risk profile. Finally, regarding cognitive ability, [10] reported that there is greater risk tolerance among people with low and high cognitive ability, showing that there is no linearity in this relationship.

These results contribute to the understanding of the risk profile of investors, however, they treat the variables in an isolated way. Moreover, among these studies only one simulated real decisions of investors, the others use questionnaires for their analysis [9]. Thus, the principal objective of this paper is to investigate which paradigm or model best portrays the risk profile manifest by investors in their financial asset investment decisions. The four paradigms used to explain this profile were: prospect theory, investor profile analysis (IPA), which is related to financial institutions’ obligation to assess an investor’s risk profile before they invest, the Big Five Personality Test, and the Cognitive Reflection Test (CRT), which measure’s people’s cognitive capacity. The choice of proxy for the investor’s risk preferences (profile) was defined by simulating investments in a laboratory setting.

Working from this starting point, the specific objectives are to: (i) assess whether people’s personalities influence their investment decisions and risk preferences; (ii) identify whether performance in the CRT might provide evidence on people’s risk behavior; and (iii) classify individuals into risk profiles according to the IPA approved by the Brazilian Association of Financial and Capital Market Entities (ANBIMA) and the International Organization of Securities Commissions (IOSCO) and administered by financial institutions, evaluating the influence of these characteristic on decision-making under risk.

This study employs the experimental method to achieve these objectives, with application of structured questionnaires, and computer simulation of investments with *Expecon* software utilizing data on real financial instruments that are available on the market [11, 12]. This makes it possible to identify respondents’ behavior in terms of their preferences for financial instruments and their risk profiles. The results are analyzed using an ordered logistic regression model.

These considerations stated, it should also be mentioned that the motivation for this study primarily comes from the importance of understanding the many different aspects that can alter risk behavior, since risk taking is a key factor that molds investments, consumption, health, and other important choices [10]. Additionally, this study is further justified by the need to better understand how behavioral and personality variables can impact on risk decisions related to investments, contributing not only to behavioral finance theory, but also to economic analyses and formulation of public policies [13].

In addition to the introduction, this paper presents a literature review about the main paradigms used in this work, the research hypotheses, the data collection, the discussion of the results and the conclusions.

## Theoretical framework

### Behavioral finances and prospect theory

For many decades, studies of people’s decision-making under uncertainty were guided by the expected utility theory [14]. According to this theory, economic behavior is seen as rational behavior. This hypothesis has been questioned and was challenged by [4], who proposed an alternative theory that they called prospect theory. This theory has become one of the most important tools used in behavioral finance to explain a series of biases affecting decision-making under conditions of risk.

An essential characteristic of this theory is that people taking decisions take into account changes to their wealth or wellbeing, rather than considering the final position. In other words, they evaluate changes or differences to their position considering a reference point, rather than evaluating absolute magnitudes. Thus, the context of the experience determines a reference point, and the stimuli are perceived in relation to this reference. This implies that the same level of wealth may seem to be a great deal to one person, but very little to another, depending on their current assets [4]. The value is therefore attributed to gains and losses and not to the final assets position.

Prospect theory suggests that people are risk averse in relation to gains and risk seeking in relation to losses. This means that the value function is S-shaped, being concave above the reference point and convex below it [4]. In general, the value function has the following characteristics: (i) it is defined in terms of displacements from a reference point; (ii) it is concave for gains and convex for losses; (iii) it is steeper for losses than for gains.

The concept of loss aversion emerges from this value function. According to this concept, people suffer more pain from loss than the pleasure they reap from an equivalent gain. Thus, the agent of behavioral finance judges gains and losses with relation to a reference point and so people exhibit risk-averse behavior with relation to gains and risk seeking with relation to losses. Agents are therefore loss-averse, since when faced with the possibility of a loss, they will accept risk in order not to realize the loss [15].

[4] contested the expected utility theory, showing evidence of patterns of behavior incompatible with the theory’s axioms. In other words, there is a pattern of behavior in which there is no evidence to support the expected utility theory, showing that errors are systematic and non-random. The expected utility theory is therefore inadequate in the majority of models of economic behavior [4, 12].

### Theories of the personality

Economists are starting to consider aspects of the personality as relevant to economic studies. [16] demonstrate the relevance of personality to the economy. Currently, the most accepted taxonomy for definition of personality is centered on the “Big five personality traits”.

The Big Five Personality Traits is the personality model that has been most widely researched and adopted [17]. This model groups personality traits under five major factors, in order to represent personality on a wide level of abstraction. It therefore suggests that differences between individuals can be classified within these five dimensions: openness to experience, conscientiousness, extraversion, agreeableness, and neuroticism ([16], p.983).

Many different instruments containing measurement items have been developed to capture personality differences. For example, the BFI-10 comprises just 10 short sentences, each of which should be assessed by the respondent against a scale ranging from "completely disagree" to "strongly agree". The responses can be used to compute a profile in each of the five major dimensions of personality.

Each individual’s personality corresponds to a combination of the five personality traits. Thus, each person can be placed on a scale in which one personality trait will be more evident than the others. This does not imply that the other traits are not also present. Thus, participants in a sample can be classified according to the predominance of characteristics as high, moderate, or low for each major dimension of personality.

#### Empirical studies of the five personality traits

A wide range of studies have been conducted to identify the influence that personality characteristics have on investment decisions, on risk taking, on decisions relating to debt, on economic preferences, and on other factors. This subsection presents some of the studies that have investigated how the big five personality traits are related to different variables, with particular attention to individuals’ risk-taking profiles.

A study by [18] based on the Big Five Personality Model analyzed the influence on investments choices of emotional stability, extraversion, risk, return, agreeableness, conscientiousness, and reasoning. Their results showed that personality has an impact on decision-making and influences choice of investment method.

[19] analyzed influences of personality traits on debt and on decisions about maintenance of financial instruments by families. High levels of the characteristics extraversion and openness to experience had a significant influence on total debt and financial instruments held, although extraversion has an inverse effect on financial asset holding. [19], therefore, concluded that there is strong evidence that personality influences aspects of people’s economic and financial decision-making.

Specifically, in relation to risk preference, [8], [20] and [21] all explored the impact of personality traits. [8] tried to identify the ways in which personal characteristics influence investors’ risk perceptions. Their results showed that people who are more extrovert tend to be especially involved in short-term investments, whereas long-term investments are preferred by those who score high for openness to experience. People who have a high score for the characteristic neuroticism were averse to short-term investments. The results revealed a significant negative correlation between the openness to experience personality trait and risk aversion. The extraversion trait was negatively related to prevention of investment risk. The characteristics extraversion and conscientiousness were positively related to short-term investment [8]. Similar results were observed by [21], who found that risk propensity was linked with high scores for extraversion and openness to experience and low scores for neuroticism, agreeableness, and conscientiousness.

Similarly, [20] examined the relationship between people’s personality traits and their economic preferences. Their results showed that the characteristic neuroticism is negatively related to risk taking in the domain of gains, but that the effect of neuroticism is reduced in the domain of losses. The characteristic conscientiousness affected attitude to risk-taking. Intelligence was also a determinant of preference for more risky options.

### Cognitive reflection test (CRT)

Countless phenomena can be associated with greater or lesser cognitive capacity, such as preference for risk, intertemporal preference, aversion to ambiguity, etc. However, the influence of people’s different cognitive capacities on their decisions has been studied little.

According to [22], possible effects of cognitive abilities (or cognitive traits) are generally part of unexplained variance in studies that specifically analyze average behavior. However, as shown by [23], intelligence, or specific cognitive abilities, are important determinants of decision-making and should not therefore be ignored.

The Big Five model captures the majority of specific personality traits. In this paper we have operationalized intellect as a separate concept to openness to experience, which is one of the components of the Big Five. This justifies combining the cognitive reflection test (CRT) with the Big Five [24]. The CRT was presented in a study by [22] and it attempts to measure people’s cognitive capacity. It is designed to assess the capacity to substitute an impulsive, and incorrect, response with reflection that leads to the correct response. The metric used to assess the relationship between CRT results and risk preferences was based on choices between a certain gain/loss and the probability of a larger gain/loss.

### Investor profile analysis

A procedure that has been adopted internationally to match investors’ investments to their risk profiles is designed to set formal standards to determine how appropriate an investment is for a customer’s risk profile. An individual’s risk profile is constructed by considering several different characteristics including their financial situation, experience with investments, risk tolerance, investment time horizon, and investment objectives, among other factors [25].

The International Organization of Securities Commissions (IOSCO) is recognized as the global organization responsible for stock market regulation, with more than 95% of the world’s stock markets affiliated, and it publishes the major guidelines for investment matching policies. The requirements are intended to afford consumers with protection, since these products have terms, resources, and investment risks that may make them difficult to understand [26].

ANBIMA (*Associação Brasileira das Entidades dos Mercados Financeiro e de Capitais*) is the principal representative organization for institutions that do business in the financial and stock markets of Brazil and, in consideration of the international guidelines, has made it obligatory for institutions that sign up to its Regulatory and Best Practices Code to analyze investors’ profiles before they invest, through adoption of an Investor Profile Analysis (IPA) process. For this study, it was decided to employ the Bank of Brazil investor profile analysis questionnaire, in view of the bank’s significant role in the country’s financial sector.

## Research hypotheses

Each individual has a risk profile that determines their behavior in the face of investment under uncertainty conditions. Thus, there are those risk-tolerant individuals who end up investing in more volatile assets and, on the other hand, there are individuals who refuse to expose themselves to risk, even with the possibility of higher returns. Such a description is easily verified in the different choices of each investor, but the determinants of this risk profile are still subject to investigation.

One of the determinants of the individual risk profile that has been explored is the personality traits. [27] investigate how personality traits can change investors' risk tolerance. The results indicate that more risk tolerant individuals are more extroverted and intuitive. Likewise, [8] found that greater openness to new experiences triggers a greater predisposition to risk and [21] show that individuals with higher neuroticism have a lower predisposition to risk in the domain of losses.

These evidences prove that there is a relation between the personality traits and the risk profile of the investors. This is justified by the alteration that these personality traits can cause in the evaluation that each economic agent realizes of the conditions of uncertainty of the market [5]. However, even if there is evidence of this relationship it is still incipient, which does not allow the definition of a clear hypothesis of the expected relationships. In this way, the hypothesis 1 is established more generally as follows:

H1: *Personality traits are determinant to consolidate the risk profile of investors*;

In line with personality traits, cognitive ability can also be a determinant of investors' risk profile. People with greater cognitive ability tend to understand the financial market more consistently and thus are able to process information more easily which expands their participation in the stock market [28]. This is more likely, according to [22] and [29], that people with greater cognitive ability tend to have a riskier profile. Thus, the second research hypothesis is established as follows:

H2: *Individuals with greater cognitive ability present greater tolerance to risk;*

Other variables that can determine investors' decisions in a risk environment are behavioral biases. Contrary to modern finance, behavioral finance understands that the choices of economic agents are not made on the basis of all available information and are not fully rational [3]. On the contrary, the errors of judgment and the cognitive biases resulting from these evaluations make the choices of economic agents to be made on the basis of a limited rationality [4].

Thus, according to [30], there is evidence that biases such as disposition effect, mental accounting, overconfidence, representativeness, restricted framework, aversion to ambiguity, anchoring, and availability bias distort the decisions of individual investors. Overconfidence and myopia were also biases flagged in another study as important to signal investment choices [9]. All of these facts confirm that there may be a violation of rationality, and the third hypothesis of this study is stated as:

H3: *The manifestation of investors' preferences is in accordance with the premises of prospect theory*.

Finally, moving away from the cognitive and behavioral aspects, this work also aims to evaluate if the questionnaires used by financial institutions are able to translate the real behavior of the economic agents. This concern has proved to be relevant, since internationally it is already confirmed that the fact that the questionnaires do not incorporate the biases inherent in investment decision-making cannot reliably portray the real behavior of economic agents in the midst of their investments. Using the Risk Tolerance Questionnaire (RTQ), [31] showed that respondents with more investment experience had more risk tolerance responses and higher risk portfolios than less experienced investors. Thus, the last hypothesis of this research is presented:

H4: *The classification of individuals into risk profiles according to the Investor Profile Analysis (IPA)*, *as recommended by the Brazilian Association of Financial and Capital Markets Entities (ANBIMA)*, *is not decisive for consolidating the real risk profile of investors;*

## Methodological procedures

This study employs the experimental method, a methodology that is relevant to the field of behavioral finance [32]. According to [33], experimental studies attempt to represent, in a simplified form, the collection of agents and institutions that make up the economy. A selection of different data collection instruments were used in the present study: several structured online questionnaires, designed to provide an understanding of risk profiles and personality, and a software package for simulating investments, used to track participants’ decisions when managing an investment portfolio.

### Data collection

The experiment was conducted with undergraduate students from the economics and electrical engineering courses at the Federal University of Santa Catarina, during modules related to finance studies. A total of 140 students took part. Thirty-four participants were women and 106 were men. However, some of them were excluded from the study because of operational problems, leaving 137 people, and the final study sample comprised the results from 124 people, since participants who stated they already knew the answers to the CRT questionnaire were also excluded.

The questionnaire was completed online at the same time by all respondents, during an experimental session. The questionnaire comprised 5 blocks of questions. The first covered aspects relative to investor profile. Next, there were 10 questions about investment scenarios, adapted from [4], to evaluate violation of the expected utility theory, and the results were used to create a dummy variable where 1 indicates that a participant predominantly (at least 6 out of 10 questions were answered in a manner compatible with prospect theory) behaved in accordance with prospect theory, while the value 0 indicates that the participant behaved in a manner compatible with expected utility theory. The third block was made up of 10 questions from the Big Five Inventory [34]. Next were a further 8 questions from the Bank of Brazil IPA questionnaire, and, finally, 3 questions from the CRT [22].

The CRT was administered separately from the other questionnaires and all participants started to answer it at the same time, because it has a time limit of 5 minutes. Once they had completed the questionnaire, all participants started the computational investment simulation at the same time. It was stressed that participation was voluntary and did not offer any type of material incentive to the participants.

It is emphasized that before the research was carried out, the project was submitted to the Brazilian “National Information System on Research Ethics involving Human Beings” (SISNEP), whose main concern is the protection of the subjects' rights. The submission to SISNEP took place via the Plataforma Brasil site, a national and unified database of research registries involving human beings. After submission, the project was evaluated by the Human Research Ethics Committee (CEP) of the Federal University of Santa Catarina. Only after the fulfillment of all the procedures and approval of the project has the research begun.

In compliance with the requirements imposed by Resolution 466/2012 of the Brazilian National Health Council (CNS), the Free and Informed Consent Form was delivered together with the questionnaire. Only those subjects who, after reading the term, agreed, in a free and clear way, to respond to the survey participated in the research. According to the Resolution, the Term of Consent guarantees, among other things, the clarification, before and during the course of the research, on the methodology used; the freedom to give up participating in the research, in any of the stages, without any kind of penalty or loss; indemnification against any damages resulting from the research; and the confidentiality of the disclosed data in order to maintain the privacy of the respondent.

### Computational investment simulation (ExpEcon)

In order to identify the respondents’ “real” behavior with relation to their preferences for assets and their appetite for risk, computational investment simulation was conducted with the aid of ExpEcon (Experimental Economics) software. This software is used to identify participants’ behavior and decisions in situations of risk [11, 12].

The assets used in the simulation were defined as those available as investment options through Bank of Brazil. Among other factors, this bank was chosen because of information contained in a classification released by ANBIMA. Their results show that Bank of Brazil tops the ranking of institutions that manage investment funds in terms of assets invested in funds. In December 2015, BB DTVM S.A had a total of R$ 591,995.8 million in assets under management in different funds, which is the largest net assets of any of the Brazilian fund managers [35]. This result illustrates the bank’s significant role in Brazil’s financial sector, justifying choosing it. Bank of Brazil investment funds were chosen with special attention to risk levels and for each risk level the fund chosen was that which had the largest net assets on the definition date, in this case, in May 2015.

The data used for simulations are real financial data from the 2006 to 2014. Half-yearly closing data were used, for which the percentage change in the asset in the corresponding 6-month period was considered. Thus, for example, period 1 of the simulator corresponds to the percentage variation of the asset in the period from January to June 2006, year 2 of the simulator corresponds to the percentage change from July to December 2006. Year 3 corresponds to the percentage change of January to June 2007 and so on. These data can be found at: https://figshare.com/s/d64e31241357afa8b950

The participants were not informed what period in time the data were from, they were only told that the data were real historic data on the assets involved. The deposit account profitability used was the six-monthly mean interest rate and was taken from the Central bank’s information system (SISBACEN), while profitability figures for Bank of Brazil investment funds were taken from the six-monthly variation per unit for each fund, taken from the Brazilian securities commission database (*Comissão de Valores Mobiliários*–CVM)

This approach to operationalization of the experiment was intended to make the simulation realistic, using historical profitability and interest data for the investment funds and deposit account. The objective of the simulation is to observe whether each participant chooses higher or lower risk assets to invest in, in order to approximate their "real" investor profile.

As input to the experimental system, all assets started period 1 with a value equal to R$ 1,000.00 and, for each subsequent year, historical real profitability was added to the initial value, thus demonstrating the evolution of the asset over the years. What was sought with this operationalization of the experiment is to make the simulation real, using real profitability data.

The participants were instructed to manage a financial investment portfolio over 18 periods. Price variations for the first 3 periods were displayed and used to provide a basis for initial investment decisions. Participants were not informed about the future performance of the assets.

The characteristics of each of the assets used in the simulation are shown in Table 1:

**Table 1:** Characteristics of assets used in the investment simulation.

Asset	Risk	Risk scale	Objective
Deposit account	Very low	0	Daily liquidity and tax free
BB Short term 50 thousand	Very low	1	Tracks CDI interbank deposit rate and is short term.
BB Fixed Income 500	Low	2	Tracks interest rate variations.
BB Fixed Income LP 50 thousand	Medium	3	Tracks interest rate variations.
BB Fixed Income LP Price Index 5 thousand	High	4	To achieve a return compatible with fixed rate investments.
BB Vale Shares	Very High	5	Made up of Vale S/A shares

Source: Bank of Brazil.

The results of portfolios were analyzed in order to capture participants’ risk profiles and thus identify their asset preferences. In this study, it was decided to employ the weighted mean of assets held in the portfolio in the last three periods during which the agent bought or sold assets. This was used to classify agents into one of three risk profiles. These risk profiles resulting from the investment simulation using ExpEcon were used to populate a variable, termed Simulator_Profile, which would be used as the dependent variable for the logistic regression model. According to the portfolio they are holding at the end of the simulation, each participant is allocated a risk profile and a number to represent it, which is the dependent variable in the model (Dummy). The low risk profile is coded with the value 1, the moderate risk profile with value 2, and the daring risk profile is coded as 3.

### Data analysis

An ordered logistic regression model was used to achieve the study objective. Table 2 contains a description of each of the variables used in this stage of the study:

**Table 2:** Descriptions of study variables.

Variables	Measurement	Description
Simulator_Profile (dependent variable)	Profile categorized on a scale from 1 to 3, based on mean risk	Classified in three risk profiles: Risk-averse agents are coded as 1, when the participant has a weighted mean risk from 0 to 2 points, agents who accept moderate risk are coded as 2, when the final weighted risk of the assets in their portfolio is 2.1 to 4.0, and the risk-seeking profile is coded as 3, when the participant is tolerant of a high level of risk, with a weighted mean from 4.1 to 6.0.
CRT Profile	Number of correct responses, on scale of 0 to 3	For each participant, this variable is equal to the number of correct answers on the Cognitive reflection test.
Mean IPA	Mean risk score	This is the participant’s mean weighted risk calculated from answers to the IPA questionnaire and the respective weightings for each response.
Mean prospect	Dummy	This dummy variable takes the value 1 when the participant predominantly behaves in a manner compatible with prospect theory, i.e., if the majority of the questionnaire items were answered according to that theory. The variable takes the value 0 if the participant predominantly behaves in a manner compatible with the alternative theory–expected utility.
Mean Personality	Mean personality profile	This variable is obtained by taking the arithmetic means of the result obtained after scoring each response to the BFI-10 questions.
Extraversion Dummy	Scale from 1 to 3	Personality profiles that that take the value 1 if the participant scores low for that dimension, 2 if the participant has a moderate score, and 3 for participants with a high score.
Agreeableness Dummy		
Conscientiousness Dummy		
Neuroticism Dummy		
Openness to experience Dummy		

Source: Data collected during study.

The logistic regression model assumes that the response variable exhibits a natural order of options. The model employs an index, with a single multinomial variable that is inherently ordered [36, 37].

According to [37], the model is constructed by starting from the same form as a multinomial logit model:

$$y^{*}=x^{\prime }\beta +\varepsilon .$$ (1)

In which, y* is not observed. What is observed is

$$\begin{array}{c} y=0,\;\mathrm{if}\;y^{*}\leq 0.\\ y=1,\;\mathrm{if}\;0<y^{*}\leq \mu_{1}.\\ y=2,\;\mathrm{if}\;\mu_{1}<y^{*}\leq \mu_{2}.\\ \vdots \\ y=J,\;\mathrm{if}\;\mu_{J-1}\leq y^{*}. \end{array}$$

Where μ_s_ is an unknown parameter, to be estimated from β. The probabilities are therefore as follows:

$$Prob(y=0|x)=\Phi (-x^{\prime }\beta )$$ (2)

$$Prob(y=1|x)=\Phi (\mu_{1}-x^{\prime }\beta )-\Phi (-x^{\prime }\beta )$$ (3)

$$Prob(y=2|x)=\Phi (\mu_{2}-x^{\prime }\beta )-\Phi (\mu_{1}-x^{\prime }\beta )$$ (4)

$$Prob(y=J|x)=1-\Phi (\mu_{J-1}-x^{\prime }\beta )$$ (5)

For probabilities to take positive values, necessarily

$$0<\mu_{1}<\mu_{2}<\cdots \mu_{J-1}$$

The function Φ(.) is a notation used for the standard normal distribution. As with other logistic regression models, the regressors’ marginal effects on the probabilities are not equal to the coefficients. However, the sign of the regression parameter can be interpreted as an increase or not of the ordered variable. Thus, if β_j_ is positive, then an increase in x_ij_ necessarily reduces its probability of being in the lowest category (y_i_ = 1) and increases the probability of being in the highest category [36].

However, according to [37], the marginal effects of the variables can be obtained from, for example, the following probabilities:

$$\frac{\partial Prob(y=0|x)}{\partial x}=-\Phi (-x^{\prime }\beta )\beta$$ (6)

$$\frac{\partial Prob(y=1|x)}{\partial x}=[\Phi (-x^{\prime }\beta )-\Phi (\mu_{1}-x^{\prime }\beta )]\beta$$ (7)

$$\frac{\partial Prob(y=2|x)}{\partial x}=\Phi (\mu -x^{\prime }\beta )\beta$$ (8)

Since the model does not illustrate a linear relationship between variables, the coefficients obtained from ordered logistic regression should not be interpreted as a direct increase of the probability. According to [37] and [36], the signs of the coefficients are unequivocal. However, it is necessary to interpret the coefficients with caution. They should be interpreted considering their marginal effects.

## Analysis of the results

The profile of the participants showed that the sample was balanced in terms of the proportion of students from each of the two undergraduate courses, with around 50.4% from the economics degree and 49.6% from the electrical engineering degree. The majority (94.2%) of the participants were single. Married students accounted for just 2.9% of the sample and those with other types of marital status also accounted for 2.9% of the sample. The majority were male (75.2%) and aged less than 25 years, since they were university students. These data are presented in Table 3.

**Table 3:** Descriptive statistics of respondents profile.

Variable	Alternatives	Percentage
Marital status	Not married	94.2%
	Married	2.9%
	Other	2.9%
Sex	Female	75.2%
	Male	24.8%
Age	Under 25 years	83.9%
	From 25 to 40 years	15.2%
	From 41 to 55 years	0.7%
	Greater than 55 years	0%

Of the variables investigated, the first result observed was the risk profile captured using the investment simulator, i.e. decision-making when faced with “real” decisions. Thus, based on their investment decision choices, 11 people (8.87%) were defined as risk averse on the system, 68 (54.84%) were identified as having moderate risk behavior, and 45 (36.29%) as having a daring risk profile. However, according to the IPA investor profile questionnaire, the sample predominantly exhibited a moderate risk tolerance profile (52.5%). The timid profile accounted for 35.7% of the sample and the daring risk profile for 11.7%.

Evaluating respondents’ behavior with respect to prospect theory, it was found that 113 participants (92%) violated expected utility theory, corroborating the assumptions of prospect theory and confirming that in situations of risk people do not take decisions compatible with expected utility theory [14]. With relation to personality traits, the findings revealed that the participants predominantly had “High” scores for personality characteristics in all dimensions and the characteristics possessed by the greatest numbers of participants were openness to experience (85%) and conscientiousness (74%). Finally, assessing cognitive capacity, it was found that just 14% of the sample answered all questions correctly, indicating elevated cognitive capacity, and 32% did not answer any of the three questions correctly.

### Ordered logistic regression model

An ordered logit model was used to evaluate behavioral and personality variables that possibly have an impact on the risk profile in “real” environments. The dependent ordered variable, the Simulator_Profile, corresponds to the 3 risk levels extracted from the investment simulation: Low, Moderate, and High risk. Analyses of the results are conducted by comparing these different categories. Thus, the coefficient is calculated maintaining the other categories constant. The initial results are shown in Table 4.

**Table 4:** Ordered logistic regression model.

Models	1	2	3	4	5
Explanatory variables	Ordered dependent variable				
	Simulator_Profile				
**Mean IPA**	4.286***	14.06***	32.96***	33.04***	35.98***
	(0.737)	(2.156)	(5.629)	(5.653)	(6.400)
**CRT Profile**		-2.875***	-5.626***	-5.648***	-6.161***
		(0.504)	(0.961)	(0.967)	(1.103)
**Mean_Prospect**			-6.729***	-6.735***	-7.096***
			(1.457)	(1.463)	(1.537)
**Mean personality**				-0.0979	-0.0175
				(0.343)	(0.457)
**Extraversion Dummy**					0.0371
					(0.353)
**Agreeableness Dummy**					-0.0202
					(0.339)
**Conscientiousness Dummy**					-0.727
					(0.473)
**Neuroticism Dummy**					-0.0825
					(0.329)
**OpennessExpDummy**					1.343**
					(0.654)
**Constant cut1**	1.744**	7.597***	6.309***	5.705**	9.017***
	(0.712)	(1.449)	(1.849)	(2.799)	(3.459)
**Constant cut2**	5.591***	13.21***	14.71***	14.14***	18.41***
	(0.910)	(1.981)	(2.816)	(3.428)	(4.406)
**R^2^**	**0.1981**	**0.4351**	**0.5907**	**0.5910**	**0.6227**
**Observations**	124	124	124	124	124

Source: Data collected during study.

(1) The table lists the coefficient for the variable with the standard deviation in parentheses.

* 10% significance

** 5% significance

*** 1% significance

Table 1 contains five different ordered logistic regression models. Each one shows the results of including additional variables and model 5 has the greatest explanatory power (R^2^ is 62.2%) and will therefore be adopted for subsequent analyses. It was found that the statistically significant variables were Mean IPA, CRT Profile, Mean Prospect, and OpennessExpDummy. The β obtained in the regression reflects the impact of changes on the probability of X, but the results are best interpreted by calculating the exact values of the probabilities [37].

For example, it is possible that, for a unit increase (from 0 to 1) in mean participant risk, calculated from their responses to the IPA questionnaire, it is expected that the probability of the participant being on a higher risk tolerance level would increase, since the respective coefficient in the fifth model is positive. This is assuming that the other variables all remain constant. This result is coherent, since both measures assess individuals’ risk tolerance.

The results for the impact that cognitive capacity has on the risk profile indicate that an increase in the number of correct answers to the CRT questionnaire (indicating greater cognitive capacity), triggers a reduction in the probability of greater risk taking. These results with relation to the CRT contradict findings reported by [22]. His study confirmed the hypothesis that participants with higher levels of education and intelligence exhibit higher risk tolerance, finding that in the domain of gains a group with High CRT was willing to risk more and to risk larger sums. [29] also reported similar findings, indicating that people with greater cognitive capacity were significantly more willing to take risks in lottery experiments.

In contrast, when [22] evaluated risk taking in the domain of losses, he found that the group that scored high on the CRT sought less risk and were more willing to accept a guaranteed loss than a probability of a loss with lower expected value. In this case, in the domain of losses, the results observed by [22] are in agreement with those observed in the present study. Similarly, [10] revealed that the relationship between risk tolerance and cognitive capacity is non-linear. They state that people classified as at the two extremes of cognitive ability, i.e., those with low cognitive capacity and those with elevated cognitive capacity have greater risk tolerance.

The results for the variable Mean_Prospect, which represents violation or compliance with expected utility theory, revealed an inverse relationship. In other words, as an individual changes from 0 to 1 (compatible with prospect theory) there is a reduction in willingness to accept risk. This result is compatible with the certainty effect. According to [4], people tend to choose certain gains over probable results, indicating loss aversion.

The OpennessExpDummy variable reflects high, medium, or low scores for this characteristic in personality dimensions. A unit increase in this variable is expected to be related to an increase in the probability that the individual will accept higher levels of risk. These results for the dimension openness to experience are in line with results observed in a study by [8], who reported that this personality trait has an inverse relationship with risk aversion, indicating that people who have the characteristics creativity and novelty seeking are willing to take greater risks. These results also point in the same direction suggested by [21], that risk tolerance is directly related with the dimension openness to experience. Thus, high scores for openness to experience indicate greater risk propensity.

Starting from these initial results, since it is known that the estimations of logistic regression models do not directly reflect marginal responses, as in the traditional method of ordinary least squares, it is necessary to analyze the marginal coefficients of each explanatory variable on the basis of the mean values for the sample. This estimation method makes it possible to calculate the marginal effects separately for each alternative (Table 5).

**Table 5:** Marginal effects of the ordered logit model for risk taking.

Variables	Alternatives		
	Averse	Moderate	Daring
**CRT Profile**	0.002 ^ns^ [0.003]	**0.807** ^***^ [0.193]	**-0.810** ^***^ [0.193]
**Mean IPA**	-0.016 ^ns^ [0.020]	**-4.719** ^***^ [1.081]	**4.735** ^***^ [1.082]
**Mean Prospect**	0.003^ns^ [0.003]	0.930^***^ [0.252]	-0.934^***^ [0.252]
**Mean personality**	7.870^ns^ [0.003]	0.002^ns^ [0.060]	-0.002^ns^ [0.060]
**Extraversion Dummy**	-0.000^ns^ [0.003]	-0.005^ns^ [0.050]	0.005^ns^ [0.050]
**Agreeableness Dummy**	7.360^ns^ [0.003]	0.002^ns^ [0.046]	-0.002^ns^ [0.046]
**Conscientiousness Dummy**	0.000^ns^ [0.000]	0.094^ns^ [0.066]	-0.094^ns^ [0.066]
**Neuroticism Dummy**	0.000^ns^ [0.000]	0.101^ns^ [0.046]	-0.101^ns^ [0.046]
**OpennessExp Dummy**	-0.000^ns^ [0.000]	-0.176^**^ [0.085]	0.176^**^ [0.086]
**LR Statistic** **Prob** **Pseudo R N.** **obs.**	140.87 0.000 0.662 124		

Note:

*** *p*≤0.01

** *p*≤0.05, * *p*≤0.10

^ns.^*p*>0.01; [] standard error.

Observing the marginal results of ordered logit regression model 5, the first finding of note is that for the Low risk tolerance level from the investment simulation, none of the variables were significant. In other words, they are not determinants of consolidation of the risk-averse profile. In contrast, for the moderate and daring profiles, the variables already discussed in relation to the results shown in Table 2 were significant.

Thus, taking the CRT profile first, it is understood that an increase in the number of correct answers on the cognitive reflection test reduced the probability of respondents exhibiting a daring risk profile on the simulator by 0.810 percentage points. However, an increase in the number of correct answers on the CRT increased the probability of participants exhibiting a moderate risk profile on the simulator by 0.807 percentage points. This confirms that, in the setting analyzed, individuals with high cognitive capacity tended not to take excessive risk in investment decisions but were not conservative and rather had a moderate profile. This result is in line with descriptions by [7], who pointed out that the relationship between these two variables is non-linear.

With relation to IPA profiles, it was found that an increase in the level of risk allocated by the IPA increased the probability that respondents would exhibit a daring risk profile on the investment simulator by 4.745 percentage points, which is coherent, since both measures provide a risk profile. With regard to the variable representing violation or compliance with expected utility theory, it was observed that an increase in compliance with prospect theory reduced the probability that respondents would behave in a risk-seeking manner in their investment decisions on the simulator by 0.934 percentage points, confirming the existence of the certainty effect and risk aversion in unstable conditions [4]. Finally, it was also of note that of the five personality traits investigated, only openness to experience proved relevant to the risk profile, where an increase in this characteristic increased the probability that individuals would exhibit a daring risk profile by 0.176 percentage points, confirming the prevailing literature [8, 21].

## Discussion

Comparing the results presented with the hypotheses proposed in the study, the findings are summarized in Table 6

**Table 6:** Summary of results achieved.

Research hypotheses	Results
H1: *Personality traits are determinant to consolidate the risk profile of investors*.	Partially accept
H2: *Individuals with greater cognitive ability present greater tolerance to risk*.	Partially accept
H3: *The manifestation of investors' preferences is in accordance with the premises of prospect theory*.	Accepted
H4: *The classification of individuals into risk profiles according to the Investor Profile Analysis (IPA)*, *as recommended by the Brazilian Association of Financial and Capital Markets Entities (ANBIMA)*, *is not decisive for consolidating the real risk profile of investors*.	Accepted

The first hypothesis was partially accepted, because only the personality trait of openness to new experiences proved to be significant. A possible justification for this result may be related to the very characteristic of the personality traits. Openness to new experiences is directly related to the individual's predisposition to change and variety [16], aspects consistent with people more prone to risk who need to be willing to make decisions that can trigger uncertain outcomes [5].

In this sense, it contributes to the understanding that cognitive ability can influence the real risk profile of individuals. As to the results, a positive relationship between cognitive ability and the risk profile was expected [28, 22]. However, the results showed the opposite. A plausible explanation for this result is based on the same argument that people with greater financial knowledge end up having greater risk aversion [38]. In the same way as financial knowledge, greater cognitive ability can cause individuals to have a greater understanding of the environment, take greater account of the risks, to better evaluate the possibilities and thus to become more cautious in their decisions and consequently present a more conservative risk profile in their investments.

In relation to the third hypothesis, it was identified that individuals effectively violate the theory of expected utility in their investment decisions incurring cognitive biases as claimed by behavioral finance [4]. This result corroborates [9] and [30], as well as the confirmation of the forth hypothesis, by pointing out the need to incorporate the analysis of behavioral bias into questionnaires that assess the risk profile of investors, enabling results that are more consistent with reality. Therefore, hypothesis four was accepted, indicating that the adopted profile questionnaire does not adequately describe the real risk profile of the investors, which makes clear the need to incorporate in these instruments all the aspects indicated in this study as determinants of the consolidation of the real risk profile.

## Conclusions

With the intention of contributing to finance studies, this article aimed to understand which of the following procedures is most relevant to understanding the true profile of an investor in situations involving decisions under risk: Investor profile analysis (IPA), prospect theory and personality theory, using the Big Five Personality Test, or the Cognitive Reflection Test (CRT).

This study employed ExpEcon software to attempt to understand the risk preferences of economic agents (in this case students). This program is designed to portray a simplified investment scenario in which participants manage an investment portfolio, buying and selling assets with different levels of risk, classified by the financial institution Bank of Brazil. The result of the simulation was used to classify participants into one of three risk profiles: risk averse, moderate risk, or bold risk (high risk tolerance).

The principal results reveal that the probability of participants exhibiting the moderate or high risk profiles on the investment simulator changes in association with changes in the investor profile obtained on the IPA, cognitive ability, compliance with prospect theory, and the personality trait openness to experience. More specifically, it was found that people’s classification according to the IPA is appropriate for understanding their risk profiles. With relation to the influence of personality on risk behavior, it was found that people who had higher scores for characteristics within this dimension (openness to experience) were more likely to take greater risk. These results are compatible with the findings of studies such as those by [8] and [21].

With regard to prospect theory, the results confirmed that increased violation of utility theory led to reduced willingness to accept risk. Finally, the total number of correct answers on the CRT exhibited the inverse behavior to risk tolerance profile, indicated by its negative coefficient. Thus, the greater the participant’s cognitive abilities, captured here by correct answers on the CRT questionnaire, the lower the probability of accepting high risk levels. This result confirms the instability in the relationship between these variables that has been identified by [10].

Also, this result differs from the existing ones, as it simultaneously assesses the impact of several variables on the real risk profile of investors and not only on the evaluation through questionnaires. In addition, these results contribute both in a theoretical manner to the consolidation of the behavioral finance area, and in a practical manner, by providing subsidies so that financial institutions can better understand the risk profile of their clients and thus propose better investment alternatives. In this sense, with regard to the analysis of the investor's profile, the evidence presented here also helps to improve the internal processes of financial institutions by seeking valid instruments to verify the level of propensity to risk of their clients.

It is not uncommon for a work with a bold objective to present limitations. The first one was the difficulty of having access to the methodology of analysis of the API questionnaire of *Banco do Brasil*, because it is an internal information to the institution. Another difficulty was in relation to the extrapolation of the results. Still in relation to the sample, it should be noted that the universe of participants was limited to undergraduate students with common characteristics, interests and degree of knowledge about similar investments and this may have generated some selection bias. Thus, if the study were to be replicated to experienced investors with a high degree of financial knowledge and diverse preferences, the results may present other evidence, which may be a possibility for future studies.

In addition to the previous recommendations, it is also suggested for future studies that the questionnaire for analysis of the investor's profile be replaced by a questionnaire from an institution with international operations, since in this study we opted for a national institution. Actually, Brazilian banks have a series of legal restrictions on establishing international operations. It is suggested the expansion of the sample to levels where the study can be generalized and extrapolated. It is suggested a randomized study sample classification, without focusing on a specific universe of agents (as in the case of this study, undergraduate students).

## Supporting information

S1 Questionnaire English. (DOCX)

S2 Questionnaire Portuguese. (DOCX)
