# Supplementary material for: Personality traits and investor profile analysis: A behavioral finance study
Source: PLoS One. 2019 Mar 27;14(3):e0214062. doi: 10.1371/journal.pone.0214062 (PMC6436746; doi:10.1371/journal.pone.0214062)
Supplement: S1 Questionnaire — (DOCX) [file pone.0214062.s001.docx]

**S1 Questionnaire**

**APPENDIX 1: Personal questionnaire**

Your Registration Number

________________________

Tick the desired option with an X:

1. Civil status
() Single () Married () Widowed () Other

2. Gender
( ) Female ( )Male

3. Indicate your current course
( ) Undergraduate in Administration ( ) Undergraduate in Economics ( ) Undergraduate in Accounting Sciences ( ) Undergraduate in Engineering ( ) Masters (any area) ( ) PhD (any area).

4. Have you ever applied money on any financial investment (Ex: stock exchange/government bonds via *Tesouro Direto*)?
( ) Yes ( ) No ( ) Do not want to answer

5. Are you aware about capital market investments?
( ) No ( ) Little ( ) Reasonably ( ) I know well ( ) I know a lot.

**APPENDIX 2: Prospect Theory Questionnaire**

**Instructions for answering the questionnaire**

The following are some scenarios of the decision-making process. Please, in each of the problems choose the alternative that you believe is the most convenient. It should be noted that the questions are similar, but it is necessary that all be answered with attention. NOTE: There is no right or wrong answer.

**Problem 1. Which of the two alternatives do you prefer?**

( ) Alternative A
33% chance of winning $ 2500
66% chance to win $ 2400

( ) Alternative B
100% chance of winning $ 2400

**Problem 2. Which of the two alternatives do you prefer?**

( ) Alternative C
33% chance of winning $ 2500
67% chance to win $ 0

( ) Alternative D
34% chance of win $ 2400
66% chance of win $ 0

**Problem 3. Which of the two alternatives do you prefer?**

( ) Alternative A
80% chance of winning $ 4000
20% chance of winning $ 0

( ) Alternative B
100% chance of winning $ 3000

**Problem 4. Which of the two alternatives do you prefer?**

( ) Alternative C
20% chance of winning $ 4000
80% chance of winning $ 0

( ) Alternative D
25% chance of winning $ 3000
75% chance of winning $ 0

**Problem 5. Which of the two alternatives do you prefer?**

( ) Alternative A
50% chance of winning a three-week trip to England, France and Italy
50% chance of not winning anything

( ) Alternative B
100% chance of winning a one-week trip to England

**Problem 6. Which of the two alternatives do you prefer?**

( ) Alternative C
5% chance of winning a three-week trip to England, France
95% chance of not winning anything

( ) Alternative D
10% chance of winning a one-week trip to England
90% chance of not winning anything

**Problem 7. Which of the two alternatives do you prefer?**

( ) Alternative A
45% chance of winning $ 6000
55% chance of winning $ 0

( ) Alternative B
90% chance of winning $ 3000
10% chance of winning $ 0

**Problem 8. Which of the two alternatives do you prefer?**

( ) Alternative C
0.1% chance of winning $ 6000
99.9% chance of winning $ 0

( ) Alternative D
0.2% chance of winning $ 3000
99.8% chance of winning $ 0

**Problem 9. Consider a two-stage game. In the first stage, there is a 75% probability that the game ends without you winning anything and a 25% chance of moving to the second stage. If you reach the second stage, you can choose between the following alternatives. Note that the choice must be made before the game starts.**

( ) Alternative A
80% chance of winning $ 4000
20% chance of winning $ 0

( ) Alternative B
100% chance of winning $ 3000

**Problem 10. In addition to the resources that you own, you received an additional $ 1000. You should now choose between the alternatives below.**

( ) Alternative A
50% chance of winning $ 1000
50% chance of winning $ 0

( ) Alternative B
100% chance of winning $ 500

**Problem 11. In addition to the resources that you own, you received an additional $ 2,000. You should now choose between the alternatives below.**

( ) Alternative C
50% chance of losing $ 1000
50% chance of losing $ 0

( ) Alternative D
100% chance of losing $ 500

**APPENDIX 3: Questionnaire to identify the personality traits.**

**Instructions for answering the questionnaire**

The following statements concern your perception about yourself in a variety of situations.

Your task is to indicate the numerical option that best expresses your opinion about yourself in each of the statements below. There are no "right" or "wrong" answers, just choose the number that you think best reflects yourself on each statement. Evaluate each statement carefully.

To do this, use the following range of responses:

| 1  I totally disagree | 2  Disagree in part | 3  Neither agree nor disagree | 4  I agree in part | 5  I totally agree |
| --- | --- | --- | --- | --- |

**I see myself as someone who:**

- **Extroversion**

Is reserved.

Is sociable, outgoing.

- **Affability**

Usually trusts people

Tends to be critical about others (finding defects)

- **Consciousness**

Tends to be lazy

Insists to complete the task or the job

- **Neuroticism**

Is relaxed, handles stress well

Gets nervous easily

- **Openness to experience**

Has little artistic interests

Has a fertile imagination

**APPENDIX 4: Cognitive Reflection Test**

Please answer the following questions:

(You have a total of 90 seconds or 30 seconds for each question)

**(1)** A bat and a ball cost $1.10 in total. The bat costs $1.00 more than the ball. How much does the ball cost?

_______ cents.

**(2)** If it takes 5 machines 5 minutes to make 5 widgets, how long would it take 100 machines to make 100 widgets?

_______ minutes

**(3)** In a lake, there is a patch of lily pads. Every day, the patch doubles in size. If it takes 48 days for the patch to cover the entire lake, how long would it take for the patch to cover half the lake?

_______ days

**Answers (Not Shown to the participant)**

(1) 5 cents

(2) 5 minutes

(3) 47 days

**Intuitive answers, but that are wrong**

(1) 10 cents

(2) 100 minutes

(3) 24 days

**APPENDIX 5: Questionnaire on Investor Profile Analysis**

**1. Indicate your age group.**a. Under 25 years
b. From 25 to 40 years
c. From 41 to 55 years
d. Above 56 years

**2. If you made a financial investment today (e.g., investment funds, savings account, treasury bonds), what percentage of your total equity would you invest in?**

a. Up to 25%

b. Between 26% and 50%

c. Between 51% and 75%

d. More than 75%

**3. When you perform any application (investment), which is the average investment time?**

a. Indefinitely since I have no plans for its use
b. A maximum of 5 years
c. A maximum of 2 years
d. 1 year maximum

**4. What is the main objective of your investments?**

a. Obtain profitability higher than that provided by traditional fixed-income investments, even assuming a risk of possible losses

b. Diversify investments

c. Form a financial reserve for future use

d. Preserve my patrimony

**5. What is your best benchmark for profitability?**

a. Stock exchange indices
b. Dollar
c. Treasury bonds
d. Savings account

**6. What financial applications do you have the most knowledge about?**

a. Mutual equity funds, stocks or derivatives
b. Multimarket funds, foreign exchange funds, foreign debt funds or fixed income funds
c. Certificate of deposit (CD), savings account or treasury bills
d. I'm not aware

**7. Have you invested in stocks or equity fund?**

a. Yes, because the possibility of greater profitability attracts me a lot
b. Yes, but with concern

c. No, but I could invest in an opportune time
d. No and I do not intend to invest, because I don’t like the idea of being subject to negative profitability

**8. If your application had a loss in the short term, what would be the acceptable percentage?**

a. Above de 15%

b. Up to 15%

c. Up to 5%

d. Would not accept losses
