# Supplementary material for: Personality traits and investor profile analysis: A behavioral finance study
Source: PLoS One. 2019 Mar 27;14(3):e0214062. doi: 10.1371/journal.pone.0214062 (PMC6436746; doi:10.1371/journal.pone.0214062)
Supplement: S2 Questionnaire — (DOCX) [file pone.0214062.s002.docx]

**S2 Questionnaire**

**Anexo 1: Questionário pessoal**

Número de Matrícula

________________________

Assinale com um X a opção desejada:

1. Estado civil

( ) Solteiro ( ) Casado ( ) Viúvo ( ) Outros

1. Gênero

( ) Feminino ( ) Masculino

1. Indique seu curso atual

( **)** Graduação em Administração ( ) Graduação em Economia ( ) Graduação em Ciências contábeis ( ) Graduação em Engenharia ( ) Mestrado (qualquer área) ( ) Doutorado (qualquer área).

# Você já aplicou dinheiro em algum investimento financeiro (Ex: bolsa de valores/Tesouro Direto)?

( ) Sim ( ) Não ( ) Não quero responder

# Tem conhecimento sobre aplicações financeiras?

( ) Nenhum ( ) Pouco ( ) Razoavelmente ( ) Conheço bem ( ) Conheço muito.

**Anexo 2: Questionário da Teoria do Prospecto**

**Instruções para responder o questionário**

A seguir apresentam-se alguns cenários do processo de tomada de decisão. Por favor, em cada um dos problemas escolha a alternativa que julgar mais conveniente. Ressalta-se que as questões são parecidas, todavia é necessário que todas sejam respondidas com atenção. OBS: não existe resposta certa ou errada.

**Problema 1. Qual das duas alternativas você prefere?**

( ) Alternativa A

33% de chances de ganhar $2500

66% de chances de ganhar $2400

( ) Alternativa B

100% de chances de ganhar $2400

**Problema 2. Qual das duas alternativas você prefere?**

( ) Alternativa C

33% de chances de ganhar $2500

67% de chances de ganhar $0

( ) Alternativa D

34% de chances de ganhar $2400

66% de chances de ganhar $0

**Problema 3. Qual das duas alternativas você prefere?**

( ) Alternativa A

80% de chances de ganhar $4000

20% de chances de ganhar $0

( ) Alternativa B

100% de chances de ganhar $3000

**Problema 4. Qual das duas alternativas você prefere?**

( ) Alternativa C

20% de chances de ganhar $4000

80% de chances de ganhar $0

( ) Alternativa D

25% de chances de ganhar $3000

75% de chances de ganhar $0

**Problema 5. Qual das duas alternativas você prefere?**

( ) Alternativa A

50% de chances de ganhar uma viagem de três semanas para a Inglaterra, França e Itália

50% de chances de não ganhar nada

( ) Alternativa B

100% de chances de ganhar uma viagem de uma semana para a Inglaterra

**Problema 6. Qual das duas alternativas você prefere?**

( ) Alternativa C

5% de chances de ganhar uma viagem de três semanas para a Inglaterra, França

95% de chances de não ganhar nada

( ) Alternativa D

10% de chances de ganhar uma viagem de uma semana para a Inglaterra

90% de chances de não ganhar nada

**Problema 7. Qual das duas alternativas você prefere?**

( ) Alternativa A

45% de chances de ganhar $6000

55% de chances de ganhar $0

( ) Alternativa B

90% de chances de ganhar $3000

10% de chances de ganhar $0

**Problema 8. Qual das duas alternativas você prefere?**

( ) Alternativa C

0,1% de chances de ganhar $6000

99,9% de chances de ganhar $0

( ) Alternativa D

0,2% de chances de ganhar $3000

99,8% de chances de ganhar $0

**Problema 9. Considere um jogo de dois estágios. No primeiro estágio, existe uma probabilidade de 75% de que o jogo termine sem que você ganhe nada e uma probabilidade de 25% de que se mova ao segundo estágio. Se você atingir o segundo estágio, você pode escolher entre as alternativas a seguir. Observe que a escolha deve ser feita antes do início do jogo.**

( ) Alternativa A

80% de chances de ganhar $4000

20% de chances de ganhar $0

( ) Alternativa B

100% de chances de ganhar $3000

**Problema 10. Além dos recursos que você possui, você recebeu mais $1000. Agora, você deve escolher entre as alternativas a seguir.**

( ) Alternativa A

50% de chances de ganhar $1000

50% de chances de ganhar $0

( ) Alternativa B

100% de chances de ganhar $500

**Problema 11. Além dos recursos que você possui, você recebeu mais $2000. Agora, você deve escolher entre as alternativas a seguir.**

( ) Alternativa C

50% de chances de perder $1000

50% de chances de perder $0

( ) Alternativa D

100% de chances de perder $500

**Anexo 3: Questionário para identificar os traços da personalidade.**

**Instruções para responder o questionário**

As declarações a seguir dizem respeito à sua percepção sobre si mesmo em uma variedade de situações.

Sua tarefa é indicar a opção numérica que melhor expressa a sua opinião sobre você mesmo, em cada uma das afirmações abaixo. Não há respostas "certas" ou "erradas", apenas escolha o número que você considera que melhor reflete você mesmo em cada declaração. Avalie cada declaração cuidadosamente.

Para isso, utilize a escala de respostas a seguir:

| 1  Discordo totalmente | 2  Discordo em parte | 3  Nem concordo nem discordo | 4  Concordo em parte | 5  Concordo totalmente |
| --- | --- | --- | --- | --- |

**Eu me vejo como alguém que:**

- **Extroversão**

É reservado.

É sociável, extrovertido.

- **Afabilidade**

Geralmente confia nas pessoas.

Tende a ser crítico com os outros (encontrar defeitos)

- **Consciência**

Tende a ser preguiçoso.

Insiste até concluir a tarefa ou o trabalho.

- **Neurotiscismo**

É relaxado, controla bem o estresse.

Fica nervoso facilmente.

- **Abertura à experiência**

Tem poucos interesses artísticos.

Tem uma imaginação fértil.

**Anexo 4: Teste de Reflexão cognitiva**

Por favor, responda às seguintes perguntas:

(Você tem um total de 90 segundos ou 30 segundos para cada pergunta)

**(1)** Um bastão e uma bola custam 1,10 dólares no total. O bastão custa US$ 1,00 mais do que a bola. Quanto custa a bola?

_______ Centavos.

**(2)** Se 5 máquinas demoram5 minutos para fazer 5 widgets, quanto tempo levariam 100 máquinas para fazer 100 Widgets?

_______ Minutos.

**(3)** Em um lago, existe uma “cobertura” de determinada planta aquática. Todos os dias, a “cobertura” dobra de tamanho. Se a “cobertura” demora 48 dias para cobrir todo o lago, quanto tempo será necessário para que a cobertura cubra metade do lago?

________ Dias

**Respostas (Não mostradas ao participante)**

(1) 5 centavos

(2) 5 minutos

(3) 47 dias

**Respostas intuitivas, mas que estão erradas**

(1) 10 centavos

(2) 100 minutos

(3) 24 dias.

**Anexo 5: Questionário Análise do Perfil do Investidor**

**1. Indique sua faixa etária.**

a. Abaixo de 25 anos

b. De 25 a 40 anos

c. De 41 a 55 anos

d. Acima de 56 anos

**2. Selecione o percentual que seus investimentos representam do total de seu patrimônio. Questão adaptada para: Se você realizasse um investimento financeiro hoje (ex: fundos de investimento, poupança, cdi), qual o percentual do total de seu patrimônio que você aplicaria em investimentos.**

a. Até 25%

b. Entre 26% e 50%

c. Entre 51% e 75%

d. Mais de 75%

**3. Quando você realiza alguma aplicação (investimento financeiro), qual o prazo médio dos investimentos?**

a. Indefinidamente, pois não tenho planos para utilização

b. No máximo 5 anos

c. No máximo 2 anos

d. No máximo 1 ano

**4. Qual o principal objetivo de seus investimentos?**

a. Obter rentabilidade superior à proporcionada por investimentos tradicionais de renda fixa, mesmo assumindo risco de possíveis perdas

b. Diversificar os investimentos

c. Formar uma reserva financeira para utilização futura

d. Preservar o patrimônio

**5. Qual a sua melhor referência de rentabilidade?**

a. Índices das Bolsas de Valores

b. Dólar

c. CDI

d. Poupança

**6. Quais são as aplicações financeiras em que você tem maior conhecimento?**

a. Fundo de Ações, Ações ou Derivativos

b. Fundos Multimercados, Cambiais, Dívida Externa ou Renda Fixa

c. CDB, Poupança ou Fundos Referenciados DI e Curto Prazo

d. Não tenho conhecimento

**7. Você já investiu em ações ou Fundo de Ações?**

a. Sim, pois a possibilidade de maiores rentabilidades me atraem muito

b. Sim, mas com receio

c. Não, mas poderia investir num momento oportuno

d. Não e não pretendo investir, pois não me agrada a idéia de estar sujeito a rentabilidade negativa

**8. Caso a sua aplicação tivesse uma perda no curto prazo, qual seria o percentual aceitável?**

a. Acima de 15%

b. Até 15%

c. Até 5%

d. Não aceitaria perda
